# Supplementary material for: Saccharomyces boulardii (CNCM I-745) improves intestinal damage in sepsis by remodeling intestinal flora structure
Source: Front Cell Infect Microbiol. 2025 Jul 31;15:1602792. doi: 10.3389/fcimb.2025.1602792 (PMC12350329; doi:10.3389/fcimb.2025.1602792)
Supplement: Supplementary file 1 [file DataSheet1.zip › summary/summary/7_Differential_analysis/1_diff_abundance/5_Family/Group.DW4_vs_CW4_vs_BW4_vs_AW4/Group.DW4_vs_CW4_vs_BW4_vs_AW4_diff_barplot.pdf]

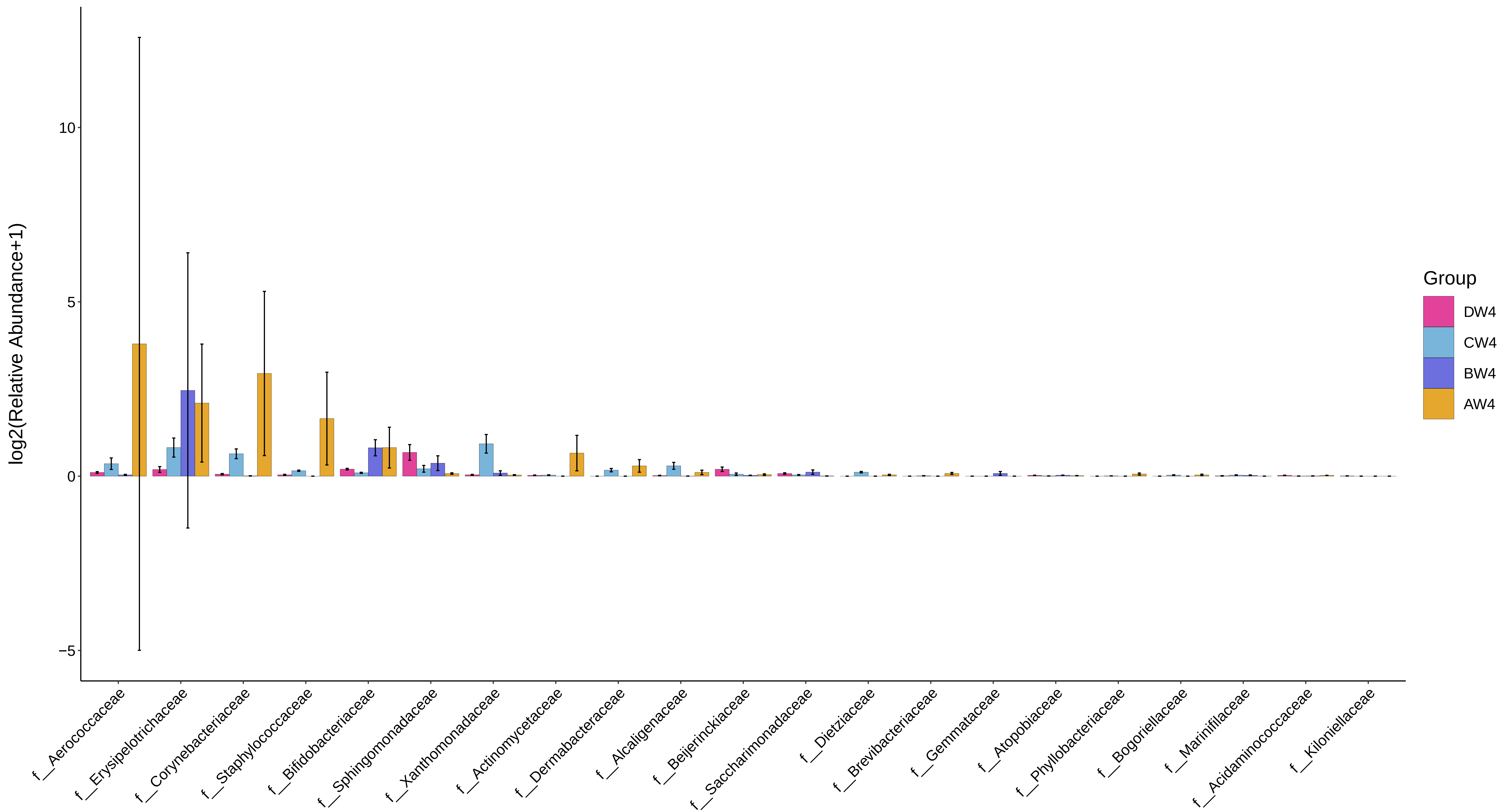

log2(Relative Abundance+1)
10
5
0
−5
f__Aerococcaceae
f__Erysipelotrichaceae
f__Corynebacteriaceae
f__Staphylococcaceae
f__Bifidobacteriaceae
f__Sphingomonadaceae
f__Xanthomonadaceae
f__Actinomycetaceae
f__Dermabacteraceae
f__Alcaligenaceae
f__Beijerinckiaceae
f__Saccharimonadaceae
f__Dietziaceae
f__Brevibacteriaceae
f__Gemmataceae
f__Atopobiaceae
f__Phyllobacteriaceae
f__Bogoriellaceae
f__Marinifilaceae
f__Acidaminococcaceae
f__Kiloniellaceae
Group
DW4
CW4
BW4
AW4
